# Impact of Western Diet and Ultra-Processed Food on the Intestinal Mucus Barrier

**DOI:** 10.3390/biomedicines11072015

**Published:** 2023-07-18

**Authors:** Carmine Stolfi, Teresa Pacifico, Giovanni Monteleone, Federica Laudisi

**Affiliations:** 1 Department of Systems Medicine, University of Rome “Tor Vergata”, 00133 Rome, Italy; carmine.stolfi@uniroma2.it (C.S.); teresa.pacifico@uniroma2.it (T.P.); gi.monteleone@med.uniroma2.it (G.M.); 2 Gastroenterology Unit, Policlinico Universitario Tor Vergata, 00133 Rome, Italy

**Keywords:** goblet cells, intestinal mucus barrier, Western diet, food additives, microbiota, ultra-processed foods, prebiotics, probiotics

## Abstract

The intestinal epithelial barrier plays a key role in the absorption of nutrients and water, in the regulation of the interactions between luminal contents and the underlying immune cells, and in the defense against enteric pathogens. Additionally, the intestinal mucus layer provides further protection due to mucin secretion and maturation by goblet cells, thus representing a crucial player in maintaining intestinal homeostasis. However, environmental factors, such as dietary products, can disrupt this equilibrium, leading to the development of inflammatory intestinal disorders. In particular, ultra-processed food, which is broadly present in the Western diet and includes dietary components containing food additives and/or undergoing multiple industrial processes (such as dry heating cooking), was shown to negatively impact intestinal health. In this review, we summarize and discuss current knowledge on the impact of a Western diet and, in particular, ultra-processed food on the mucus barrier and goblet cell function, as well as potential therapeutic approaches to maintain and restore the mucus layer under pathological conditions.

## 1. Introduction

In healthy people, the intestinal tract has the largest population of immune cells in the body, reflecting the fact that its surface is continuously exposed to food antigens and a complex and dynamic population of microorganisms—called “gut microbiota”—which encompasses bacteria, fungi, protists, archaea, and viruses [1,2]. This state of “physiological” inflammation faces the challenge of responding quickly to pathogens without inducing morphological/functional intestinal changes [3]. Therefore, complex and highly regulated immune responses by different types of mucosal cells must maintain intestinal immune homeostasis in a healthy intestinal tract [3]. The intestinal epithelium acts as a semipermeable physical barrier that facilitates the absorption of essential nutrients, commensal-derived metabolites, electrolytes, and water from the intestinal lumen into the bloodstream [4]. Each intestinal epithelial cell maintains strict contact with its neighbors and seals the surface of the intestinal tract with tight junctions [4]. Furthermore, the intestinal epithelium contains goblet cells that, by secreting mucins, are key to the establishment of the first line of defense against pathogens (that is, the mucus barrier) [5] and Paneth cells, which synthesize antimicrobial peptides (i.e., lysozyme and defensins) [6]. However, luminal antigens can reach mucosal immune cells through the follicle-associated epithelium, which overlies the organized lymphoid tissues of the intestinal wall (i.e., Peyer’s patches and follicles) [7]. Furthermore, mucosal dendritic cells can extend processes across intestinal epithelial cells into the lumen to sample bacteria [8,9]. Although luminal antigens interact with the intestinal immune system, excessive adaptive responses are prevented by additional counter-regulatory mechanisms, including suppression of lymphocytes by regulatory T cells, regulatory macrophages/dendritic cells, immune-suppressive cytokines (e.g., IL-10), and induction of programmed T cell death by apoptosis [3].

The gut microbiota exerts a marked influence on the host during normobiosis, and altered gut microbiota composition (dysbiosis), characterized by a decrease in microbiota diversity, a loss of beneficial microbiota, or an overgrowth of harmful microbiota, has been associated with the pathogenesis of many inflammatory diseases, such as Crohn’s disease and ulcerative colitis, and infections [10,11].

Dysbiosis can arise from host-specific conditions such as genetic background, health status (e.g., infections, inflammation, psychological and physical stress), and lifestyle habits (e.g., poor hygiene) or, more importantly, can be driven by environmental factors such as exposure to xenobiotics (e.g., antibiotics, drugs, food additives, alcohol, and nicotine) [12,13,14].

In addition to the factors mentioned above, diet represents one of the main elements that can influence gut homeostasis. For example, high-fat and high-sugar diets, which are commonly present in westernized countries, can trigger intestinal inflammation, whereas high-fiber intake can limit or prevent such detrimental effects [15].

In this review, we summarize and discuss current knowledge on the impact of the Western diet on the mucus barrier and goblet cell function, as well as potential therapeutic approaches to maintain and restore the mucus layer under pathological conditions.

## 2. Western Diet and Ultra-Processed Food

The typical Western diet pattern is characterized by a high intake of saturated fatty acids, sugar, refined carbohydrates, protein, and salt, as well as a low amount of fiber and fruits [15]. Most Western diet-related food products undergo a transformation process that generates formulations of ingredients typically created by a series of industrial techniques and processes, often including the use of food additives and specific cooking methods, called ultra-processed food (UPF) [16,17,18]. Furthermore, UPF intake has been associated with higher host levels of exogenous advanced glycation end products (AGEs), which are harmful compounds generated when sugars react with proteins and lipids during fast and high-temperature cooking [18,19,20]. Interestingly, several clinical studies are underway to assess the potential harmful effects of these compounds on intestinal health and the possible correlation with the onset and development of inflammatory bowel disease (IBD) [21,22].

The direct (Table 1) and indirect (Table 2) impacts of UPF and natural dietary products on the function/activity of the mucus barrier and goblet cells are summarized and discussed below.

### 2.1. Direct Effects of the Western Diet, Ultra-Processed Food, and Natural Dietary Products on the Intestinal Mucus Barrier

#### 2.1.1. High-Protein Diet

Several reports in recent years indicated that a high protein content could have important detrimental effects on the mucus barrier. For example, in vivo studies showed that rats exposed to a high-protein diet (53% protein) for two weeks developed important goblet cell hyperplasia and a higher ileal mucus content, together with increased expression of mucin (Muc)-2 and Muc-3 proteins [23]. Milder effects were observed in the colon, where the number of goblet cells in the epithelium decreased and was balanced by a higher number of those located in the colonic crypts, suggesting that the high-protein diet influenced the differentiation process of colonic epithelial cells in their transition from the crypt to the epithelial surface instead of mucus production [23].

Except for mild alterations in some immunological parameters in the ileal mucosa (that is, decreased expression of interleukin (IL)-1β, -4, -10, -17A, interferon (IFN)-γ, and tumor necrosis factor (TNF)-α levels, as well as increased expression of IL-13 and transforming growth factor (TGF)-β) and mesenteric lymph nodes (that is, decreased frequency of CD3^+^ CD4^+^ T cells), the high-protein diet did not exert any further effect on other components, such as luminal levels of IgA and expression of antimicrobial peptides [23]. The authors hypothesized that increased expression of the *Il-13* gene may be partially responsible for the increase in ileal mucus content, as IL-13 has been proposed to contribute to the differentiation and hyperplasia of goblet cells in other biological systems [49,50]. However, the fact that the level of IL-13 protein was almost undetectable [23] strongly suggests the involvement of other factors in such an effect. An alternative hypothesis could be that the high-protein diet provides a large amount of amino acids, in particular threonine, serine, and proline, which are essential for the structure of mucins, supporting previous observations that indicated the beneficial effects of oral administration of L-threonine, L-serine, L-proline, and L-cysteine on mucus production during experimental colitis [51].

#### 2.1.2. High-Fat Diet

As already discussed, one of the main characteristics of the Western diet is a large intake of foods rich in saturated fatty acids, which critically affect gut homeostasis and microbiota composition, thus triggering low-grade inflammation over time. Importantly, a high-fat diet can also affect intestinal epithelial cells, in particular goblet cells, without any microbiota-mediated mechanisms. For example, Gulhane and colleagues observed that mice exposed to a high-fat diet for 22 weeks presented an aberrant mucus layer and a reduction in colonic mucus content due to the induction of chronic endoplasmic reticulum (ER) stress in goblet cells by non-esterified long-chain saturated fatty acids [24]. In particular, a high-fat diet triggered the gene expression of *Grp78* and IRE1β (*Ern2*), an ER stress sensor that is expressed specifically in the ER membrane of goblet cells, where it promotes efficient protein folding and mucin secretion [52]. This resulted in a decrease in Muc-2 and Klf-4 expression, a marker of goblet cell terminal differentiation, already after 11 weeks of treatment, together with the accumulation of misfolded proteins and non-O-glycosylated Muc-2 precursors [24].

#### 2.1.3. Plant Polyphenols

The mucus content can also be influenced by the dietary plant polyphenols available in some foods and beverages (that is, apples, tea, and coffee). For example, chlorogenic acid, epicatechin gallate, and quercetin were found to modulate the gene expression of some mucins (e.g., *Muc-3, -13, -17*, and, to a lesser extent, *Muc-2*) in Caco-2/HT29-MTX cocultures, although these results were not confirmed at the protein levels [25]. Another interesting plant polyphenol is resveratrol, which has been reported to exert antineoplastic effects in some animal models of cancer [53]. In particular, in a rat model of colorectal cancer, resveratrol has been shown to upregulate *Muc-2* expression in colon tissue once administered for 30 weeks, together with a suppressive effect on several neoplastic markers (e.g., cyclooxygenase-2, ornithine decarboxylase), although the mechanism underlying the beneficial effects of resveratrol on Muc-2 content was not investigated [26].

#### 2.1.4. Food Additives

Food additives are widely present in UPFs because they play an important role in maintaining and improving quality and taste [54]. Several studies revealed that some of them can directly modulate mucus production and goblet cell physiology. For example, our group found that maltodextrin (MDX), a polysaccharide commonly used as a thickening agent during food processing, could trigger ER stress in normal goblet cells and exacerbate experimental intestinal inflammation [27]. In detail, colonocytes of mice exposed for four weeks to MDX (5% *w*/*v*) dissolved in drinking water strongly upregulated IRE-1β and showed a marked reduction in glycosylated Muc-2 and total mucus content through a mechanism mediated by mitogen-activated protein kinase (MAPK) p38 [27]. Interestingly, the level of Muc-2 mRNA increased as a compensatory mechanism for the decrease in the amount of mature Muc-2 protein, suggesting that these alterations would be mainly due to impaired Muc-2 folding and secretion rather than defects in goblet cell differentiation and function [27]. Inhibition of ER stress was able to restore mucus maturation and secretion by goblet cells and limit intestinal inflammation in a mouse model of experimental colitis, while no other effects were reported on other components of the epithelial barrier (i.e., tight junctions) as well as on the mucosa-associated microbiota [27].

Some of the results were confirmed by Zangara et al. in IL-10 knockout (KO) mice [55], which are known to spontaneously develop colitis and showed an easier penetrable mucus layer and aberrant colonic Muc-2 synthesis since IL-10 can prevent unresolved ER stress in goblet cells and associated protein misfolding [56]. Once fed MDX, mice developed colitis earlier than controls and showed reduced mucus production and increased phosphorylation of p38 MAPK. However, MDX-exposed IL-10-KO mice also revealed an altered microbiota composition in the caecum, characterized by decreased levels of acetic acid and increased epithelial proliferation, as well as increased expression of the intracellular microbial sensor NOD2 [55]. The authors also reported a direct effect of MDX on goblet cells, whose number was reduced in treated animals, together with altered expression of the transcription factor Klf4, even if this last observation was limited only to in vitro experiments [55].

Nanoparticles (NPs) are an important category of food additives that are often used during food processing as a result of their coloring and antimicrobial properties. Among them, silver nanoparticles (Ag-NPs, E174) and titanium dioxide nanoparticles (TiO_2_-NPs, E171) are particularly present in many products and food packaging, thus entering directly or indirectly into contact with the consumer [57,58]. Several studies revealed that Ag-NPs and TiO_2_-NPs exerted a relevant effect on microbiota composition and could easily be trapped by the mucus layer to contain their spread across the epithelial barrier [58]. However, chronic in vitro exposure of intestinal epithelial cells to TiO_2_-NPs may also result in oxidative stress and DNA damage [29]. Interestingly, an in vivo study by Ruiz and colleagues reported a worsening of the intestinal inflammation in mice undergoing experimental colitis when treated orally with TiO_2_-NPs, which accumulated in intestinal epithelial cells, spleen, and macrophages and activated the NLRP3 inflammasome [30]. However, no mucus layer alterations have been reported [30]. The in vivo effects of Ag-NPs were also evaluated. Male and female rats showed a higher number of ileal mucus-secreting goblet cells when orally exposed to different doses of Ag-NPs (30, 300, and 1000 mg/kg/day) for 28 weeks, whereas no effect was observed in the proximal colon [31]. Furthermore, such treatment induced alterations in mucus composition characterized by a decreased content of neutral and acidic mucins in the ileum and in the whole colon/rectum [31]. Additional in vivo observations confirmed that Ag-NPs can target the mucus layer by affecting the expression of the *Muc-2* and *Muc-3* genes in the ileal mucosa, especially in female rats orally exposed to different doses of Ag-NPs (9, 18, and 36 mg/kg/day) for 13 weeks [32].

#### 2.1.5. AGEs

Ultra-processed food typically undergoes different industrial transformations that also include specific cooking methods (i.e., dry heating techniques such as grilling or frying), leading to the production of advanced glycation end products (AGEs). Furthermore, the intake of non-digestible carbohydrates can also produce toxic glycating metabolites during fermentation, thus contributing to the release of AGEs [33]. In addition to their stimulating effect on mast cells, AGEs generated by lactose and fructo-oligosaccharide intake for three weeks in C57BL/6 mice induced an increase in mucus production as a consequence of increased discharge of goblet cells into the empty distal colon in response to mucosal irritation, as well as an altered thickness of the mucus layer covering feces [33]. Such effects were reverted by the action of the antiglycation agent pyridoxamine, suggesting that the generation of AGEs during digestion greatly affects the mucus barrier [33]. The authors hypothesized that AGEs generated during fermentation may promote increased mast cell frequency and activation, which interact with goblet cells and trigger their dysregulated mucus discharge [33].

### 2.2. Indirect Effects of the Western Diet, Ultra-Processed Food, and Natural Dietary Products on the Intestinal Mucus Barrier

#### 2.2.1. Low-Fiber Diet

Low fiber intake is known to have a profound impact on the gut microbiota [59]. Although mucus digestion is part of physiological turnover, uncontrolled degradation by microbes exerts detrimental effects on the intestinal epithelial barrier that may, in some cases, result in decreased thickness of the inner mucus layer, increased mucus permeability, and pathogen penetration [59]. In mice, such defects may be limited by microbiota transplantation from animals fed a high-fiber diet to those exposed to a low-fiber diet (mimicking the Western diet), as it can restore the inner mucus layer through a microbiota-mediated mechanism [36]. In fact, dietary fibers can provide several polysaccharides that are not metabolized by the host but that represent a key source of nutrients for commensals [34]. Microbes produce several enzymes called harbor glycosyl hydrolases, which cleave specific glycan linkages and depolymerize and ferment dietary polysaccharides into host-absorbable short-chain fatty acids [60]. Using a gnotobiotic mouse model, Desai and co-workers transplanted a synthetic human gut microbiota, composed of fully sequenced commensal bacteria, by oral gavage into germ-free mice [35]. The authors observed that the microbial community of mice exposed to a chronic or intermittent fiber-deficient diet began to use the mucus layer rich in glycoproteins of the host as an alternative energy source. This metabolic switch affected both the composition and thickness of the mucus layer, leading to a proliferation of mucus-degrading bacteria that allowed the translocation of pathogens across the epithelial barrier and resulted in the induction of intestinal inflammation and increased susceptibility to colitis [35]. In particular, this effect was associated with a rapid increase in the abundance of *Akkermansia muciniphila* and *Bacteroides caccae* at the expense of fiber-degrading species [35]. Similar observations were reported in a mouse model of diet-induced obesity, where a high intake of saturated fats but a low level of dietary fiber (thus mimicking the Western diet) triggered important alterations in the composition of the microbiota, characterized by low levels of *Bifidobacterium longum*, which has been reported to regulate mucus thickness and penetrability [36]. In particular, the colonic inner mucus layer was affected, whereas mucus production by goblet cells in the crypts was increased as a compensatory response from the host. Fecal transplantation from mice fed normal chow to those receiving Western diet-mimicking chow was sufficient to prevent mucus defects, as was the administration of *Bifidobacterium longum* or the prebiotic inulin, which is known to sustain its growth [36].

#### 2.2.2. High-Protein Diet

The Western diet is also characterized by high protein intake, which can influence the growth of some bacterial species at the expense of others. In this regard, a recent study by Chen et al. showed that mice fed three different types of high-protein diet (HPD) (i.e., high-casein, whey, or soy protein diet) for four weeks developed more severe experimental colitis compared to controls [37]. Interestingly, a diet rich in casein triggered the growth of several mucus-degrading microbial species, especially *Bacteroides thetaiotaomicron*, in the colonic mucus layer, which impaired its thickness. Of note, treatment with an antibiotic cocktail was able to prevent a change in microbiota composition, as well as mucus dysfunction [37].

#### 2.2.3. Food Additives

In addition to their direct effects on goblet cell function and mucus secretion, some food additives can also affect the colonic mucus layer by altering the composition of the gut microbiota. Dietary emulsifiers, which are commonly used in food processing as detergent-like molecules, play an important role in this regard. In particular, Chassaing and colleagues provided several pieces of evidence on the detrimental effects of low doses of the emulsifiers polysorbate-80 (P80) and carboxymethylcellulose (CMC, E466) on the host’s bacterial community [38]. The authors first observed that wild-type, toll-like receptor (TLR)-5-KO and IL-10-KO mice exposed to 1% (*w*/*v*) P80 or 1% (*w*/*v*) CMC in drinking water or chow for 12 weeks showed increased levels of mucus-degrading and inflammatory bacteria (e.g., *Ruminococcus gnavus*, *Akkermansia muciniphila*) that compromised mucus thickness and triggered low-grade inflammation and metabolic syndrome [38]. Germ-free mice did not show such alterations, whereas they began to develop similar features once transplanted with feces derived from mice treated with CMC or P80, suggesting that changes in microbiota composition after chronic exposure to emulsifiers are sufficient to trigger low-grade inflammation and metabolic syndrome [38]. Using the mucosal simulator of the human intestinal microbial ecosystem (M-SHIME) model that simulates the human intestinal microbial community in the absence of a live host, the authors also confirmed the detrimental effects of CMC and P80 on the human microbiota [39]. Both P80 and CMC directly affected the microbiota, increasing its pro-inflammatory potential [39]. Furthermore, germ-free mice that received the microbiota treated with CMC and P80 developed low-grade inflammation and metabolic syndrome, suggesting that these food additives play a central role in mediating their detrimental effects on the host [39]. Contrasting results were obtained in mice who received a natural food emulsifier, glycerol monolaurate (150 mg/kg), for eight weeks, as it induced intestinal inflammation and metabolic syndrome but lowered the frequency of *Akkermansia muciniphila* [40]. Finally, mice exposed to different isomers of the seaweed-derived polysaccharide carrageenan (20 ng/mL in drinking water) for six weeks showed an altered gut microbiota characterized by a lower abundance of *Akkermansia muciniphila* and spontaneous colitis [41].

#### 2.2.4. AGEs

In addition to having direct detrimental effects on mucus composition, advanced glycation end products (AGEs) can also indirectly affect mucus content and colon permeability by altering the composition of the microbiota. In particular, Sprague-Dawley rats exposed to a high AGE diet for 6, 12, or 18 weeks showed reduced α-diversity, altered crypts, goblet cell depletion, dysregulated expression of the colonic tight junctions zonulin-1 and occludin-1, and increased intestinal permeability, especially in the group fed a high AGE diet for 18 weeks [42]. In line with these findings, Nie et al. reported that an AGE-enriched diet affected intestinal barrier integrity and microbial composition in C57BL/6 mice, while treatment with prebiotic galactooligosaccharides (GOS) limited these alterations by decreasing the frequency of *Akkermansia muciniphila* and increasing the number of goblet cells, tight junction expression, and short-chain fatty acid levels [43]. Furthermore, mice transplanted with fecal content from the AGE-treated group developed an altered mucus barrier, while those receiving feces from animals treated with GOS were protected [43].

#### 2.2.5. Dietary Compounds and Immune-Mediated Effects

Dietary compounds and related metabolites can also influence the host’s immune response, which is known to play a key role in maintaining the mucus layer and the function of goblet cells [61]. For example, fermentable fiber inulin has been reported to prevent alterations in microbial composition and the development of metabolic syndrome induced by exposure to a high-fat diet or in animal models of type 1 diabetes by triggering the expression of the IL-22 cytokine by type 3 innate lymphoid cells [44,45]. IL-22 is known to play an important role in mucosal healing due to its beneficial effects on epithelial regeneration by stimulating stem cell proliferation, the release of anti-microbial peptides, and the production of membrane mucus [62]. Multi-omics analysis in the distal small intestine of weaning mice showed that IL-22 could also exert important beneficial effects on membrane-bound Muc-17, which is one of the main components of the glycocalyx covering enterocytes in mice and humans [63]. In particular, IL-22 promoted Muc-17 expression in the ileal enterocytes of weaning mice to protect them from exogenous bacteria and molecules [63].

The Aryl hydrocarbon Receptor (AhR) is a ligand-activated transcription factor capable of interacting with xenobiotic molecules and dietary metabolites [64,65]. These latter are present in food, such as fruits and cruciferous vegetables, and include flavonoids and indoles, while some nutritional AhR ligands, instead, can be produced endogenously upon tryptophan catabolism by commensals (i.e., *Lactobacillus reuteri* or bacteria expressing tryptophanase) [66]. These nutritional agonists can bind to AhRs and promote the maintenance of some immune cell subsets, such as intestinal intraepithelial lymphocytes and type 3 innate lymphoid cells, as well as the differentiation of regulatory T cells [67]. The AhR is also capable of targeting intestinal epithelial cells, regulating the differentiation of intestinal crypt stem cells into enterocytes and goblet cells [68]. Interestingly, transgenic mice overexpressing the tryptophan metabolizing enzyme indoleamine 2,3-dioxygenase 1 (IDO1) in intestinal epithelial cells showed an increase in the mucus layer thickness and an increase in the frequency of secretory cells, including goblet cells [69]. In particular, IDO1 was shown to interact with AhRs to suppress Notch1 signaling [69]. Furthermore, IDO1 expression was found to be positively correlated with *Muc-2* and *AhR* gene expression in the ileum of CD patients [69]. Additional studies showed that metabolites derived from the Western diet could affect AhR signaling and alter the mucus barrier. For example, Liu et al. observed that mice exposed to a high-fat diet for two weeks produced high levels of deoxycholic acid, a secondary bile acid capable of exerting cytotoxic effects in intestinal crypts by altering IDO1 expression and tryptophan metabolism, and this effect was associated with impaired differentiation of intestinal stem cells into goblet cells and Muc-2 production [46]. It should be noted that the administration of AhR agonists, such as 6-formylindolo [3,2-b] carbazole (FICZ), partially restored AhR signaling and intestinal stem cell differentiation [46]. The interaction of FICZ with the AhR could also protect against goblet cell depletion and decreased mucus production during experimental colitis by promoting goblet cell differentiation through the AhR-p-ERK 1/2-mediated mechanism [47]. Consistently, the lack of *Ahr* gene expression in transgenic mice significantly impaired the mucus layer [47].

Another important cytokine involved in maintaining the mucus barrier is IL-10, which has been reported to protect goblet cells from ER stress by preventing the accumulation of misfolded Muc-2 in the ER and allowing its correct O-glycosylation and secretion [28]. The Western diet has been suggested to weaken these IL-10-mediated beneficial effects, as IL-10 expression and the downstream JAK-STAT pathway (that is, STAT3) were reduced in serum and adipose tissue of 62 obese children with hypertriglyceridemia and in high-fat diet-induced obese rats [48]. Unfortunately, alterations in goblet cell frequency and mucus production were not investigated or characterized in these subjects, nor was a possible direct or indirect role of IL-10 in the processes [48].

## 3. Therapeutic Improvement of the Mucus Barrier

**Figure 1:**
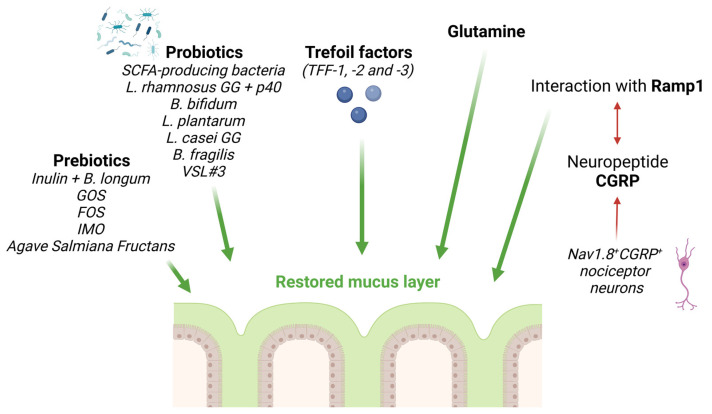
Overview of potential strategies aimed at stimulating mucus production and/or maintaining the integrity of the mucus layer. Abbreviations: *B. Longum*: *Bifidobacterium longum*; GOS: galactooligosaccharide; FOS: oligofructose; IMO: iso-malto oligosaccharide; SCFA: short-chain fatty acid; *L. rhamnosus*: *Lactobacillus rhamnosus*; *B. bifidum*: *Bifidobacterium bifidum*; *L. plantarum*: *Lactobacillus plantarum*; *L. casei GG*: *Lactobacillus casei GG*; *B. fragilis: Bacteroides fragilis*; TFF: trefoil factor; Ramp1: receptor activity modifying protein 1; CGRP: calcitonin gene-related peptide. Created with Biorender.com.

In addition to a fiber-enriched diet or the administration of AhR agonists, other strategies represent potentially valid alternatives to help maintain a stable and functional intestinal mucus layer (Figure 1).

### 3.1. Prebiotics

Prebiotics are commonly defined as non-digestible dietary elements (typically fibers) that are fermented by microorganisms and can support the growth and activity of beneficial bacteria [70]. We have already described the effect of inulin on the growth of *Bifidobacterium longum*, which is important in protecting the mucus layer from the detrimental effects of a high-fat diet, thus maintaining the content and quality of mucus [36]. Among prebiotics, galactooligosaccharide was found to impact the microbiota composition and increase Muc-2 production in old mice, generally showing altered intestinal homeostasis [71]. In line with these results, prebiotic fructooligosaccharides prevented the detrimental effects of the high-fat diet on the mucus barrier and intestinal homeostasis [72]. In detail, fructooligosaccharides promoted goblet cell differentiation (by increasing *Math1*, *Spdef*, *Elf3*, and *Klf4* gene expression), the amount of transmembrane and secreted mucins, and the expression of glycosyltransferases, which are important enzymes involved in mucin glycosylation [72]. Similar results were recently obtained after treating rats with low doses of Agave salmiana-derived fructans (5, 10, and 12.5%) for 35 days, which increased mucus production and the frequency of goblet cells [73]. Finally, isomalto-oligosaccharide has been reported to prevent abnormalities induced by a high-fat diet in the mouse colon by positively affecting mucus production, intestinal permeability, and intestinal bacterial abundance [74,75].

### 3.2. Probiotics

Another potential therapeutic intervention includes probiotics, defined as a group of special bacterial strains used as a supplement to restore the intestinal microbiota. Probiotics can also provide fermented metabolites that can support epithelial cells and restore the mucus barrier [70]. For example, bacteria producing short-chain fatty acids (i.e., *Lactobacillus rhamnosus GG*, *Bifidobacterium longum*, and *Bifidobacterium bifidum*) have attracted special attention due to their ability to stimulate mucus production and maintain the integrity of the mucus layer [36,76,77,78]. In this regard, butyric acid, a fermented product of the bacteria mentioned above, was found to be crucial for mucin gene expression and mucus production in vitro and in vivo [79,80,81,82,83]. Consistent results were obtained using other short-chain fatty acids, such as propionate and acetate, which have been reported to exert important beneficial effects on the mucus barrier [83]. Other mechanisms have been proposed to mediate the beneficial effects of specific probiotics on the intestinal barrier and mucus layer. Wang et al. showed that p40, a soluble protein derived from *Lactobacillus rhamnosus GG*, transactivated the epidermal growth factor receptor (EGFR) and the downstream target protein kinase B (AKT), which is known to regulate mucus production [76]. In particular, p40 promoted Muc-2 expression in vitro as well as mucus production in the colonic epithelium of mice orally treated with this protein (10 µg in pectin/zein beads) for four hours or five consecutive days, while no effects were observed in Egfrwa5 (EGFR dominant negative) transgenic mice [76]. Zhang and colleagues demonstrated that the administration of an adequate amount of *Bacteroides fragilis* strain ZY-312 to rats who underwent antibiotic-associated diarrhea could repair the structure of the intestinal barrier by enhancing goblet cell proliferation and the expression of tight junction proteins (i.e., ZO-1 and occludin) [84]. Furthermore, the probiotic strain *Lactobacillus plantarum 299v* was reported to promote the expression of Muc-2 and Muc-3 in vitro, thus affecting the adhesion of the *Escherichia coli* pathogen to the mucus barrier [85]. Similar effects were also observed in Caco-2 cells, used as a model of the intestinal epithelial barrier, stimulated with *Lactobacillus casei GG* [86], while conflicting results were obtained by using the probiotic mix VSL#3 (*Lactobacillus acidophilus*, *Lactobacillus bulgaricus*, *Lactobacillus casei*, *Lactobacillus plantarum*, *Bifidobacterium brevis*, *Bifidobacterium infantis*, *Bifidobacterium longum*, and *Streptococcus salivarius* subsp. *thermophilus*). For example, *Muc-2* gene expression and colonic mucus secretion have been reported to be induced in rats orally treated with a mixture of VSL#3 for seven consecutive days, and in vitro experiments in colonic epithelial cells LS174T confirmed these observations [87]. Gaudier and co-workers, instead, reported that VSL#3 was able to modify the microbiota composition in mice undergoing experimental colitis, but such an effect was not sufficient to decrease the inflammatory response, promote mucosal healing, or restore the mucus barrier [88].

### 3.3. Other Therapeutic Approaches

Trefoil factors (TFFs) are a group of peptides capable of repairing the gastrointestinal mucosa and restoring or maintaining intestinal homeostasis [89,90,91]. Although human TFF1 and TFF2 are expressed primarily by goblet cells in the stomach and duodenum, TFF3 is produced in the rest of the small intestine and colon [89,90,91]. TFF3 can interact with and bind to the glycoproteins present in the mucus, thus maintaining the mucus barrier and promoting epithelial cell migration to sustain mucosal healing during colitis [92,93,94]. TFF-mediated therapeutic effects have been tested in vivo in wild-type mice undergoing acute colitis induced by dextran sodium sulfate and in IL-10-KO mice developing chronic enterocolitis [95]. In particular, an engineered bacterium, *Lactococcus lactis*, capable of synthesizing and secreting bioactive murine TFFs, was administered by intragastric catheter to allow the same distribution of TFFs in the small intestine, caecum, and colon of mice for five consecutive days [95]. Treatment with TFFs protected against intestinal inflammation-mediated epithelial damage, including goblet cell dysfunction, in both experimental models [95].

The amino acid glutamine is a major substrate utilized by intestinal cells and has proven to be essential for the maintenance of intestinal structure and function [96]. Glutamine has been shown to promote mucus synthesis and alleviate damage to the intestinal mucus barrier after burn injury in rats [97]. Further studies from the same group linked this effect with the ability of glutamine to increase Muc-2 maturation through the enhancement of G6PD glycosylation and inhibition of AGR2 S-glutathionylation [98].

Finally, since the gastrointestinal tract is innervated by several enteric neurons that affect intestinal cell functions (e.g., motility, pain, and nutrient absorption) [99], a possible approach to improving the functionality of the mucus barrier may rely on the modulation of such cells. Yang et al. reported that mucus secretion could be stimulated by Nav1.8^+^CGRP^+^ nociceptor neurons, which are located near goblet cells [100]. In particular, the authors observed that goblet cells express receptor activity modifying protein 1 (Ramp1), which interacts with the neuropeptide calcitonin gene-related peptide (CGRP), whose delivery is regulated by nociceptors after sensing commensal bacteria and dietary stimuli [100]. The interaction of CGRP and Ramp1 stimulated colonic mucus production, thus maintaining the integrity of the colonic mucus barrier and protecting against experimental colitis [100].

## 4. Discussion

The mucus barrier represents the first line of defense against luminal pathogens to maintain gut homeostasis [101]. In particular, the small intestine is characterized by a discontinuous mucus layer, while colonic mucus is organized in an outer loose and thicker layer colonized by commensals, together with an inner sterile layer that protects epithelial cells from pathogen penetration, thus avoiding unwanted immune responses [102,103]. The role of the mucus barrier is crucial because different pathologies, such as ulcerative colitis, are characterized by important alterations of the outer and inner layers, from which a lot of detrimental effects are generated, such as microbial dysbiosis, infection, and an uncontrolled inflammatory immune response.

Environmental agents, such as diet and elements involved in food processing, can further worsen this condition. As mentioned above, a large body of experimental evidence raised concerns about the possible deleterious effects of ultra-processed food (UPF) on the function/activity of goblet cells and the intestinal barrier, and recent clinical studies would seem to support this notion. Chen and colleagues published the results of a prospective and cross-sectional cohort study of 187,854 individuals in the United Kingdom using 24-hour dietary recall questionnaires [21]. Ultra-processed food intake was higher in patients with inflammatory bowel disease (IBD) compared to controls and was associated with the incidence of Crohn’s disease, but not ulcerative colitis, and the need for IBD-related surgery [21]. Similar results were obtained from another prospective cohort study conducted with 116,087 participants from seven different regions (e.g., Europe, North and South America, Africa, the Middle East, South Asia, South East Asia, and China), who received a questionnaire on the frequency of food and were prospectively followed at least every three years [104]. The results obtained confirmed that a higher intake of UPF (such as soft drinks and refined, sweetened foods) was associated with an increased risk of incident IBD [104].

Conflicting results were obtained from another prospective cohort study conducted on 413,590 participants from eight European countries, where the association between the degree of food processing and the risk of IBD was investigated [22]. Interestingly, the risk of developing Crohn’s disease within 13 years was lower in people who ate mainly unprocessed/minimally processed foods, although no association was detected between UPF consumption and the risk of IBD [22].

However, more studies are necessary to deeply characterize the positive or negative correlation between UPF intake and the onset and development of IBD, as well as to identify the contributing factors within UPFs that are able to trigger chronic intestinal inflammation.

Despite all the promising experimental and preclinical findings, changing eating habits is just part of the advice given by clinicians, and the beneficial effects of such intervention may vary among patients. In fact, some of those affected by chronic inflammatory conditions (i.e., patients with stricturing Crohn’s disease) may not be eligible for therapeutic nutritional management characterized, for example, by increased fiber intake, as they may face several side effects such as intestinal obstruction, a lower micronutrient absorption rate, and a lack of fermentative microbe activities. Furthermore, the unwanted inflammatory response could be enhanced [105,106,107], suggesting that these subjects should be treated with alternative strategies.

In this context, the experimental data produced in vitro and in preclinical models suggest that prebiotics and probiotics are useful tools for the treatment of patients with impaired mucus barrier functionality and chronic intestinal inflammation, as well as for the prevention of these pathological conditions. In support of this view are the results of a recent systematic review by Zheng et al., where the authors conducted a meta-analysis of data from a total of 26 randomized controlled trials (n = 1891), revealing that probiotics could improve intestinal barrier function and alleviate inflammation and microbial dysbiosis [108].

## 5. Conclusions

Although there is still much to do, awareness of the potential detrimental impact of UPF on intestinal homeostasis is rising among people. In this context, health authorities are making several efforts to limit the use of UPF in the community from childhood (www.fao.org/publications/card/en/c/CA5644EN/; www.fda.gov/food/food-ingredients-packaging/food-additives-petitions) (accessed on 27 April 2023) in order to avoid a further increase in patients affected by chronic inflammatory conditions in the near future. In addition to that, research efforts to discover/validate the beneficial effects of new and/or modified microbial entities (e.g., next-generation probiotics) on preventing gut dysbiosis and/or re-establishing intestinal homeostasis are worth pursuing.

## Figures and Tables

**Table 1:** Direct impact of the Western diet, ultra-processed food, or natural dietary products on the intestinal mucus barrier.

Component	Model System	Effects on the Mucus Barrier	Reference
HPD	Rats exposed for 2 weeks (53% protein content)	Goblet cell hyperplasia, higher ileal mucus content, and increased expression of Muc-2 and Muc-3 proteins. Decreased number of goblet cells in the epithelium, balanced by a higher frequency of those in the colonic crypts. Higher expression of the *Il-13* gene.	[23]
HFD	Mice exposed for 22 weeks (46% saturated fat, 20% protein, and 4.80% crude fiber)	Aberrant mucus layer and decreased colonic mucus content. ER stress in goblet cells (increased expression of Grp78 and IRE-1β). Decreased Muc-2 and Klf4 expression.	[24]
Chlorogenic acid, epicatechin gallate, and quercetin (*plant polyphenols*)	Coculture of Caco-2/HT29-MTX (10 µM)	Increased expression of Muc-2, -3, -13, and -17.	[25]
Resveratrol (*plant polyphenol*)	CRC animal model (30 weeks, 8 mg/kg)	Upregulation of Muc-2 expression in colon tissue.	[26]
MDX (*food additive*)	Mice exposed for 4 weeks (5% MDX)	Upregulation of IRE-1β expression and reduction in glycosylated Muc-2 and total mucus content through a p38 MAPK-mediated mechanism. Increased Muc-2 mRNA levels. Exacerbated experimental colitis in treated mice.	[27]
	NOD2-KO and IL-10-KO mice exposed for 11 weeks (1% MDX)	Exacerbated experimental colitis in treated mice. Reduced number of goblet cells and mucus production, and increased phosphorylation of p38 MAPK. Altered caecal microbiota composition. Increased NOD2 expression.	[28]
TiO_2_-NPs (*food additive*)	Caco-2 cells (1–200 µg/mL)	Oxidative stress and DNA damage.	[29]
	Mice exposed for 8 days (50 or 500 mg/kg/day TiO_2_)	Exacerbated experimental colitis. Reduced mucus production and increased activation of the NLRP3 inflammasome.	[30]
Ag-NPs (*food additives*)	Rats exposed for 28 weeks (30, 300, or 1000 mg/kg/day Ag-NPs)	Higher number of ileal-secreting mucus goblet cells. Decreased content of neutral and acidic mucins in the ileum and in the whole colon/rectum.	[31]
	Rats exposed for 13 weeks (9, 18, and 36 mg/kg/day Ag-NPs)	Altered expression of the *Muc-2* and *Muc-3* genes in the ileal mucosa.	[32]
AGEs derived from lactose and FOS	Mice exposed for 3 weeks (5 mg/day lactose and 10% FOS)	Higher discharge of goblet cells in the empty distal colon. Altered thickness of the mucus layer that covers the feces. Increased frequency of mast cells.	[33]

Pink background = detrimental impact; green background = protective impact. Abbreviations: HPD: high-protein diet; MUC: mucin; IL: interleukin; HFD: high-fat diet; ER: endoplasmic reticulum; GRP78: 78-kDa glucose-regulated protein; IRE1β: Inositol-requiring enzyme 1β; KLF4: Krüppel-like factor 4; MDX: maltodextrin; MAPK: mitogen-activated protein kinase; NOD2: nucleotide-binding oligomerization domain-containing protein 2; TiO_2_ NPs: titanium dioxide nanoparticles; NLRP3: NLR family pyrin domain containing 3; Ag NPs: silver nanoparticles; AGEs: advanced glycation end products; FOS: oligofructose.

**Table 2:** Indirect impact of the Western diet, ultra-processed food, or natural dietary products on the intestinal mucus barrier.

Component	System Model	Effects on the Mucus Barrier	Reference
High-fiber diet	Fecal transplantation in mice	Restoration of the inner mucus layer.	[34]
Fiber-deficient diet	Germ-free mice transplanted with synthetic human gut microbiota and exposed to a chronic or intermittent fiber-deficient diet (40 weeks, starch and maltodextrin replaced with glucose).	Increase in mucus-degrading bacteria and decrease in fiber-degrading species. Altered mucus composition and thickness. Increased intestinal inflammation and increased susceptibility to colitis.	[35]
High saturated fats + low dietary fiber	Mouse model of diet-induced obesity (40.6% kcal of fat: 41% saturated, 52% monounsaturated, 7% polyunsaturated fatty acids)	Low levels of *B. longum*. Altered inner colonic mucus layer and increased mucus production by goblet cells in the crypts.	[36]
HPD	Mice exposed to a high-casein, whey protein, or soy protein (593 g/kg) diet for 4 weeks	Increase in mucus-degrading bacteria and exacerbated experimental colitis.	[37]
P80 and CMC (*food additives*)	Wild-type, TLR5-KO, and IL-10-KO mice exposed for 12 weeks (1% P80 and CMC)	Increase in inflammatory and mucus-degrading bacteria. Reduced mucus thickness and increased low-grade inflammation and metabolic syndrome.	[38]
P80 and CMC (*food additives*)	Microbial ecosystem (M-SHIME) model	Altered expression of flagella-encoding genes and those related to the inflammatory response.	[39]
Glycerol monolaurate (*food additive*)	Mice exposed for 8 weeks (150 mg/kg)	Lower abundance of *A. muciniphila*, intestinal inflammation, and metabolic syndrome.	[40]
Carrageenan (*food additive*)	Mice exposed to different isomers for 6 weeks (20 ng/mL)	Lower abundance of *A. muciniphila* and spontaneous colitis.	[41]
AGEs	Mice exposed for 6, 12, and 18 weeks	Reduced α-diversity, altered crypts, goblet cell depletion, dysregulated expression of zonulin-1 and occludin-1, and increased intestinal permeability.	[42]
	Mice exposed to a high-AGE diet or transplanted with feces from AGE-treated animals	Impaired mucus barrier and microbial composition, and depletion of goblet cells.	[43]
Inulin	Mice exposed to HFD (60% fat)	Protection against alterations in the mucosal barrier, microbial composition, and the development of HFD-induced metabolic syndrome by triggering the production of IL-22.	[44,45]
Deoxycholic acid (*secondary bile acid*)	Mice exposed to HFD for 2 weeks (60% fat)	Cytotoxic effects in the intestinal crypts and barrier dysfunction by altering IDO-1 expression, AhR signaling, and decreased levels of goblet cells and Muc-2.	[46]
FICZ (*AhR agonist*)	Mice treated daily with FICZ (1 µg/mouse) 2 days after induction of DSS-colitis	Differentiation of goblet cells during experimental colitis through the AhR-p-ERK1/2-mediated mechanism.	[47]
HFD	Obese children with hypertriglyceridemia and HFD-induced obese rats (44.9% fat)	Reduction in the IL-10 and JAK-STAT pathways. The effects on the mucus barrier were not characterized.	[48]

Pink background = detrimental impact; green background = protective impact; gray background = uncharacterized impact. Abbreviations: HPD: high-protein diet; P80: polysorbate 80; CMC: carboxymethyl cellulose; TLR: toll-like receptor; IL: interleukin; AGEs: advanced glycation end products; HFD: high-fat diet; IDO: Indoleamine-pyrrole 2,3-dioxygenase; AHR: aryl hydrocarbon receptor; MUC: mucin; FICZ: 6-Formylindolo[3,2-b]carbazole; p-ERK: phosphorylated extracellular signal-regulated kinase; JAK: Janus kinase; STAT: signal transducer and activator of transcription.

## Data Availability

No new data were created or analyzed in this study. Data sharing is not applicable to this article.

## References

[1] Thursby E., Juge N. (2017). Introduction to the human gut microbiota. Biochem. J..

[2] Campbell C., Kandalgaonkar M.R., Golonka R.M., Yeoh B.S., Vijay-Kumar M., Saha P. (2023). Crosstalk between Gut Microbiota and Host Immunity: Impact on Inflammation and Immunotherapy. Biomedicines.

[3] MacDonald T.T., Monteleone I., Fantini M.C., Monteleone G. (2011). Regulation of homeostasis and inflammation in the intestine. Gastroenterology.

[4] Peterson L.W., Artis D. (2014). Intestinal epithelial cells: Regulators of barrier function and immune homeostasis. Nat. Rev. Immunol..

[5] Paone P., Cani P.D. (2020). Mucus barrier, mucins and gut microbiota: The expected slimy partners?. Gut.

[6] Bevins C.L., Salzman N.H. (2011). Paneth cells, antimicrobial peptides and maintenance of intestinal homeostasis. Nat. Rev. Microbiol..

[7] Neutra M.R., Mantis N.J., Kraehenbuhl J.P. (2001). Collaboration of epithelial cells with organized mucosal lymphoid tissues. Nat. Immunol..

[8] Niess J.H., Brand S., Gu X., Landsman L., Jung S., McCormick B.A., Vyas J.M., Boes M., Ploegh H.L., Fox J.G. (2005). CX3CR1-mediated dendritic cell access to the intestinal lumen and bacterial clearance. Science.

[9] Rescigno M., Urbano M., Valzasina B., Francolini M., Rotta G., Bonasio R., Granucci F., Kraehenbuhl J.P., Ricciardi-Castagnoli P. (2001). Dendritic cells express tight junction proteins and penetrate gut epithelial monolayers to sample bacteria. Nat. Immunol..

[10] Qiu P., Ishimoto T., Fu L., Zhang J., Zhang Z., Liu Y. (2022). The Gut Microbiota in Inflammatory Bowel Disease. Front. Cell. Infect. Microbiol..

[11] Maciel-Fiuza M.F., Muller G.C., Campos D.M.S., do Socorro Silva Costa P., Peruzzo J., Bonamigo R.R., Veit T., Vianna F.S.L. (2023). Role of gut microbiota in infectious and inflammatory diseases. Front. Microbiol..

[12] Stolfi C., Maresca C., Monteleone G., Laudisi F. (2022). Implication of Intestinal Barrier Dysfunction in Gut Dysbiosis and Diseases. Biomedicines.

[13] Hrncir T. (2022). Gut Microbiota Dysbiosis: Triggers, Consequences, Diagnostic and Therapeutic Options. Microorganisms.

[14] Martinez J.E., Kahana D.D., Ghuman S., Wilson H.P., Wilson J., Kim S.C.J., Lagishetty V., Jacobs J.P., Sinha-Hikim A.P., Friedman T.C. (2021). Unhealthy Lifestyle and Gut Dysbiosis: A Better Understanding of the Effects of Poor Diet and Nicotine on the Intestinal Microbiome. Front. Endocrinol..

[15] Statovci D., Aguilera M., MacSharry J., Melgar S. (2017). The Impact of Western Diet and Nutrients on the Microbiota and Immune Response at Mucosal Interfaces. Front. Immunol..

[16] Elizabeth L., Machado P., Zinocker M., Baker P., Lawrence M. (2020). Ultra-Processed Foods and Health Outcomes: A Narrative Review. Nutrients.

[17] Monteiro C.A., Cannon G., Levy R.B., Moubarac J.C., Louzada M.L., Rauber F., Khandpur N., Cediel G., Neri D., Martinez-Steele E. (2019). Ultra-processed foods: What they are and how to identify them. Public. Health Nutr..

[18] Nie C., Li Y., Qian H., Ying H., Wang L. (2022). Advanced glycation end products in food and their effects on intestinal tract. Crit. Rev. Food Sci. Nutr..

[19] Snelson M., Lucut E., Coughlan M.T. (2022). The Role of AGE-RAGE Signalling as a Modulator of Gut Permeability in Diabetes. Int. J. Mol. Sci..

[20] Teodorowicz M., van Neerven J., Savelkoul H. (2017). Food Processing: The Influence of the Maillard Reaction on Immunogenicity and Allergenicity of Food Proteins. Nutrients.

[21] Chen J., Wellens J., Kalla R., Fu T., Deng M., Zhang H., Yuan S., Wang X., Theodoratou E., Li X. (2022). Intake of ultra-processed foods is associated with an increased risk of Crohn’s disease: A cross-sectional and prospective analysis of 187,154 participants in the UK Biobank. J. Crohn’s Colitis.

[22] Meyer A., Dong C., Casagrande C., Chan S.S.M., Huybrechts I., Nicolas G., Rauber F., Levy R.B., Millett C., Oldenburg B. (2022). Food Processing and Risk of Crohn’s Disease and Ulcerative Colitis: A European Prospective Cohort Study. Clin. Gastroenterol. Hepatol..

[23] Lan A., Andriamihaja M., Blouin J.M., Liu X., Descatoire V., Desclee de Maredsous C., Davila A.M., Walker F., Tome D., Blachier F. (2015). High-protein diet differently modifies intestinal goblet cell characteristics and mucosal cytokine expression in ileum and colon. J. Nutr. Biochem..

[24] Gulhane M., Murray L., Lourie R., Tong H., Sheng Y.H., Wang R., Kang A., Schreiber V., Wong K.Y., Magor G. (2016). High Fat Diets Induce Colonic Epithelial Cell Stress and Inflammation that is Reversed by IL-22. Sci. Rep..

[25] Volstatova T., Marchica A., Hroncova Z., Bernardi R., Doskocil I., Havlik J. (2019). Effects of chlorogenic acid, epicatechin gallate, and quercetin on mucin expression and secretion in the Caco-2/HT29-MTX cell model. Food Sci. Nutr..

[26] Sengottuvelan M., Deeptha K., Nalini N. (2009). Influence of dietary resveratrol on early and late molecular markers of 1,2-dimethylhydrazine-induced colon carcinogenesis. Nutrition.

[27] Laudisi F., Di Fusco D., Dinallo V., Stolfi C., Di Grazia A., Marafini I., Colantoni A., Ortenzi A., Alteri C., Guerrieri F. (2019). The Food Additive Maltodextrin Promotes Endoplasmic Reticulum Stress-Driven Mucus Depletion and Exacerbates Intestinal Inflammation. Cell. Mol. Gastroenterol. Hepatol..

[28] Hasnain S.Z., Tauro S., Das I., Tong H., Chen A.C., Jeffery P.L., McDonald V., Florin T.H., McGuckin M.A. (2013). IL-10 promotes production of intestinal mucus by suppressing protein misfolding and endoplasmic reticulum stress in goblet cells. Gastroenterology.

[29] Dorier M., Beal D., Marie-Desvergne C., Dubosson M., Barreau F., Houdeau E., Herlin-Boime N., Carriere M. (2017). Continuous in vitro exposure of intestinal epithelial cells to E171 food additive causes oxidative stress, inducing oxidation of DNA bases but no endoplasmic reticulum stress. Nanotoxicology.

[30] Ruiz P.A., Moron B., Becker H.M., Lang S., Atrott K., Spalinger M.R., Scharl M., Wojtal K.A., Fischbeck-Terhalle A., Frey-Wagner I. (2017). Titanium dioxide nanoparticles exacerbate DSS-induced colitis: Role of the NLRP3 inflammasome. Gut.

[31] Jeong G.N., Jo U.B., Ryu H.Y., Kim Y.S., Song K.S., Yu I.J. (2010). Histochemical study of intestinal mucins after administration of silver nanoparticles in Sprague-Dawley rats. Arch. Toxicol..

[32] Williams K., Milner J., Boudreau M.D., Gokulan K., Cerniglia C.E., Khare S. (2015). Effects of subchronic exposure of silver nanoparticles on intestinal microbiota and gut-associated immune responses in the ileum of Sprague-Dawley rats. Nanotoxicology.

[33] Kamphuis J.B.J., Reber L., Eutamene H., Theodorou V. (2022). Increased fermentable carbohydrate intake alters colonic mucus barrier function through glycation processes and increased mast cell counts. FASEB J..

[34] Sonnenburg E.D., Sonnenburg J.L. (2014). Starving our microbial self: The deleterious consequences of a diet deficient in microbiota-accessible carbohydrates. Cell Metab..

[35] Desai M.S., Seekatz A.M., Koropatkin N.M., Kamada N., Hickey C.A., Wolter M., Pudlo N.A., Kitamoto S., Terrapon N., Muller A. (2016). A Dietary Fiber-Deprived Gut Microbiota Degrades the Colonic Mucus Barrier and Enhances Pathogen Susceptibility. Cell.

[36] Schroeder B.O., Birchenough G.M.H., Stahlman M., Arike L., Johansson M.E.V., Hansson G.C., Backhed F. (2018). Bifidobacteria or Fiber Protects against Diet-Induced Microbiota-Mediated Colonic Mucus Deterioration. Cell Host Microbe.

[37] Chen L., Wang J., Yi J., Liu Y., Yu Z., Chen S., Liu X. (2021). Increased mucin-degrading bacteria by high protein diet leads to thinner mucus layer and aggravates experimental colitis. J. Gastroenterol. Hepatol..

[38] Chassaing B., Koren O., Goodrich J.K., Poole A.C., Srinivasan S., Ley R.E., Gewirtz A.T. (2015). Dietary emulsifiers impact the mouse gut microbiota promoting colitis and metabolic syndrome. Nature.

[39] Chassaing B., Van de Wiele T., De Bodt J., Marzorati M., Gewirtz A.T. (2017). Dietary emulsifiers directly alter human microbiota composition and gene expression ex vivo potentiating intestinal inflammation. Gut.

[40] Jiang Z., Zhao M., Zhang H., Li Y., Liu M., Feng F. (2018). Antimicrobial Emulsifier-Glycerol Monolaurate Induces Metabolic Syndrome, Gut Microbiota Dysbiosis, and Systemic Low-Grade Inflammation in Low-Fat Diet Fed Mice. Mol. Nutr. Food Res..

[41] Shang Q., Sun W., Shan X., Jiang H., Cai C., Hao J., Li G., Yu G. (2017). Carrageenan-induced colitis is associated with decreased population of anti-inflammatory bacterium, Akkermansia muciniphila, in the gut microbiota of C57BL/6J mice. Toxicol. Lett..

[42] Qu W., Yuan X., Zhao J., Zhang Y., Hu J., Wang J., Li J. (2017). Dietary advanced glycation end products modify gut microbial composition and partially increase colon permeability in rats. Mol. Nutr. Food Res..

[43] Nie C., Xie X., Liu H., Yuan X., Ma Q., Tu A., Zhang M., Chen Z., Li J. (2023). Galactooligosaccharides ameliorate dietary advanced glycation end product-induced intestinal barrier damage in C57BL/6 mice by modulation of the intestinal microbiome. Food Funct..

[44] Zou J., Chassaing B., Singh V., Pellizzon M., Ricci M., Fythe M.D., Kumar M.V., Gewirtz A.T. (2018). Fiber-Mediated Nourishment of Gut Microbiota Protects against Diet-Induced Obesity by Restoring IL-22-Mediated Colonic Health. Cell Host Microbe.

[45] Zou J., Reddivari L., Shi Z., Li S., Wang Y., Bretin A., Ngo V.L., Flythe M., Pellizzon M., Chassaing B. (2021). Inulin Fermentable Fiber Ameliorates Type I Diabetes via IL22 and Short-Chain Fatty Acids in Experimental Models. Cell Mol. Gastroenterol. Hepatol..

[46] Liu L., Xu J., Xu X., Mao T., Niu W., Wu X., Lu L., Zhou H. (2022). Intestinal Stem Cells Damaged by Deoxycholic Acid via AHR Pathway Contributes to Mucosal Barrier Dysfunction in High-Fat Feeding Mice. Int. J. Mol. Sci..

[47] Yin J., Yang K., Zhou C., Xu P., Xiao W., Yang H. (2019). Aryl hydrocarbon receptor activation alleviates dextran sodium sulfate-induced colitis through enhancing the differentiation of goblet cells. Biochem. Biophys. Res. Commun..

[48] Liu Y., Xu D., Yin C., Wang S., Wang M., Xiao Y. (2018). IL-10/STAT3 is reduced in childhood obesity with hypertriglyceridemia and is related to triglyceride level in diet-induced obese rats. BMC Endocr. Disord..

[49] Kondo M., Tamaoki J., Takeyama K., Nakata J., Nagai A. (2002). Interleukin-13 induces goblet cell differentiation in primary cell culture from Guinea pig tracheal epithelium. Am. J. Respir. Cell Mol. Biol..

[50] Tukler Henriksson J., Coursey T.G., Corry D.B., De Paiva C.S., Pflugfelder S.C. (2015). IL-13 Stimulates Proliferation and Expression of Mucin and Immunomodulatory Genes in Cultured Conjunctival Goblet Cells. Invest. Ophthalmol. Vis. Sci..

[51] Faure M., Mettraux C., Moennoz D., Godin J.P., Vuichoud J., Rochat F., Breuille D., Obled C., Corthesy-Theulaz I. (2006). Specific amino acids increase mucin synthesis and microbiota in dextran sulfate sodium-treated rats. J. Nutr..

[52] Cloots E., Simpson M.S., De Nolf C., Lencer W.I., Janssens S., Grey M.J. (2021). Evolution and function of the epithelial cell-specific ER stress sensor IRE1beta. Mucosal Immunol..

[53] Ko J.H., Sethi G., Um J.Y., Shanmugam M.K., Arfuso F., Kumar A.P., Bishayee A., Ahn K.S. (2017). The Role of Resveratrol in Cancer Therapy. Int. J. Mol. Sci..

[54] Laudisi F., Stolfi C., Monteleone G. (2019). Impact of Food Additives on Gut Homeostasis. Nutrients.

[55] Zangara M.T., Ponti A.K., Miller N.D., Engelhart M.J., Ahern P.P., Sangwan N., McDonald C. (2022). Maltodextrin Consumption Impairs the Intestinal Mucus Barrier and Accelerates Colitis Through Direct Actions on the Epithelium. Front. Immunol..

[56] Schwerbrock N.M., Makkink M.K., van der Sluis M., Buller H.A., Einerhand A.W., Sartor R.B., Dekker J. (2004). Interleukin 10-deficient mice exhibit defective colonic Muc2 synthesis before and after induction of colitis by commensal bacteria. Inflamm. Bowel Dis..

[57] Lomer M.C., Thompson R.P., Powell J.J. (2002). Fine and ultrafine particles of the diet: Influence on the mucosal immune response and association with Crohn’s disease. Proc. Nutr. Soc..

[58] Vitulo M., Gnodi E., Meneveri R., Barisani D. (2022). Interactions between Nanoparticles and Intestine. Int. J. Mol. Sci..

[59] Makki K., Deehan E.C., Walter J., Backhed F. (2018). The Impact of Dietary Fiber on Gut Microbiota in Host Health and Disease. Cell Host Microbe.

[60] El Kaoutari A., Armougom F., Gordon J.I., Raoult D., Henrissat B. (2013). The abundance and variety of carbohydrate-active enzymes in the human gut microbiota. Nat. Rev. Microbiol..

[61] Zheng D., Liwinski T., Elinav E. (2020). Interaction between microbiota and immunity in health and disease. Cell Res..

[62] Patnaude L., Mayo M., Mario R., Wu X., Knight H., Creamer K., Wilson S., Pivorunas V., Karman J., Phillips L. (2021). Mechanisms and regulation of IL-22-mediated intestinal epithelial homeostasis and repair. Life Sci..

[63] Layunta E., Javerfelt S., Dolan B., Arike L., Pelaseyed T. (2021). IL-22 promotes the formation of a MUC17 glycocalyx barrier in the postnatal small intestine during weaning. Cell Rep..

[64] Hankinson O. (1995). The aryl hydrocarbon receptor complex. Annu. Rev. Pharmacol. Toxicol..

[65] Mulero-Navarro S., Fernandez-Salguero P.M. (2016). New Trends in Aryl Hydrocarbon Receptor Biology. Front. Cell Dev. Biol..

[66] Scott S.A., Fu J., Chang P.V. (2020). Microbial tryptophan metabolites regulate gut barrier function via the aryl hydrocarbon receptor. Proc. Natl. Acad. Sci. USA.

[67] Ye J., Qiu J., Bostick J.W., Ueda A., Schjerven H., Li S., Jobin C., Chen Z.E., Zhou L. (2017). The Aryl Hydrocarbon Receptor Preferentially Marks and Promotes Gut Regulatory T Cells. Cell Rep..

[68] Metidji A., Omenetti S., Crotta S., Li Y., Nye E., Ross E., Li V., Maradana M.R., Schiering C., Stockinger B. (2018). The Environmental Sensor AHR Protects from Inflammatory Damage by Maintaining Intestinal Stem Cell Homeostasis and Barrier Integrity. Immunity.

[69] Alvarado D.M., Chen B., Iticovici M., Thaker A.I., Dai N., VanDussen K.L., Shaikh N., Lim C.K., Guillemin G.J., Tarr P.I. (2019). Epithelial Indoleamine 2,3-Dioxygenase 1 Modulates Aryl Hydrocarbon Receptor and Notch Signaling to Increase Differentiation of Secretory Cells and Alter Mucus-Associated Microbiota. Gastroenterology.

[70] Sanders M.E., Merenstein D.J., Reid G., Gibson G.R., Rastall R.A. (2019). Probiotics and prebiotics in intestinal health and disease: From biology to the clinic. Nat. Rev. Gastroenterol. Hepatol..

[71] Arnold J.W., Roach J., Fabela S., Moorfield E., Ding S., Blue E., Dagher S., Magness S., Tamayo R., Bruno-Barcena J.M. (2021). The pleiotropic effects of prebiotic galacto-oligosaccharides on the aging gut. Microbiome.

[72] Paone P., Suriano F., Jian C., Korpela K., Delzenne N.M., Van Hul M., Salonen A., Cani P.D. (2022). Prebiotic oligofructose protects against high-fat diet-induced obesity by changing the gut microbiota, intestinal mucus production, glycosylation and secretion. Gut Microbes.

[73] Andrade A.I.C., Bautista C.R., Cabrera M.A.R., Guerra R.E.S., Chavez E.G., Ahumada C.F., Lagunes A.G. (2019). Agave salmiana fructans as gut health promoters: Prebiotic activity and inflammatory response in Wistar healthy rats. Int. J. Biol. Macromol..

[74] Singh D.P., Singh S., Bijalwan V., Kumar V., Khare P., Baboota R.K., Singh P., Boparai R.K., Singh J., Kondepudi K.K. (2018). Co-supplementation of isomalto-oligosaccharides potentiates metabolic health benefits of polyphenol-rich cranberry extract in high fat diet-fed mice via enhanced gut butyrate production. Eur. J. Nutr..

[75] Wang W., Xin H., Fang X., Dou H., Liu F., Huang D., Han S., Fei G., Zhu L., Zha S. (2017). Isomalto-oligosaccharides ameliorate visceral hyperalgesia with repair damage of ileal epithelial ultrastructure in rats. PLoS ONE.

[76] Wang L., Cao H., Liu L., Wang B., Walker W.A., Acra S.A., Yan F. (2014). Activation of epidermal growth factor receptor mediates mucin production stimulated by p40, a Lactobacillus rhamnosus GG-derived protein. J. Biol. Chem..

[77] Capurso L. (2019). Thirty Years of Lactobacillus rhamnosus GG: A Review. J. Clin. Gastroenterol..

[78] Yoshihara T., Oikawa Y., Kato T., Kessoku T., Kobayashi T., Kato S., Misawa N., Ashikari K., Fuyuki A., Ohkubo H. (2020). The protective effect of Bifidobacterium bifidum G9-1 against mucus degradation by Akkermansia muciniphila following small intestine injury caused by a proton pump inhibitor and aspirin. Gut Microbes.

[79] Finnie I.A., Dwarakanath A.D., Taylor B.A., Rhodes J.M. (1995). Colonic mucin synthesis is increased by sodium butyrate. Gut.

[80] Gaudier E., Jarry A., Blottiere H.M., de Coppet P., Buisine M.P., Aubert J.P., Laboisse C., Cherbut C., Hoebler C. (2004). Butyrate specifically modulates MUC gene expression in intestinal epithelial goblet cells deprived of glucose. Am. J. Physiol. Gastrointest. Liver Physiol..

[81] Hamer H.M., Jonkers D.M., Vanhoutvin S.A., Troost F.J., Rijkers G., de Bruine A., Bast A., Venema K., Brummer R.J. (2010). Effect of butyrate enemas on inflammation and antioxidant status in the colonic mucosa of patients with ulcerative colitis in remission. Clin. Nutr..

[82] Hatayama H., Iwashita J., Kuwajima A., Abe T. (2007). The short chain fatty acid, butyrate, stimulates MUC2 mucin production in the human colon cancer cell line, LS174T. Biochem. Biophys. Res. Commun..

[83] Shimotoyodome A., Meguro S., Hase T., Tokimitsu I., Sakata T. (2000). Short chain fatty acids but not lactate or succinate stimulate mucus release in the rat colon. Comp. Biochem. Physiol. A Mol. Integr. Physiol..

[84] Zhang W., Zhu B., Xu J., Liu Y., Qiu E., Li Z., Li Z., He Y., Zhou H., Bai Y. (2018). Bacteroides fragilis Protects Against Antibiotic-Associated Diarrhea in Rats by Modulating Intestinal Defenses. Front. Immunol..

[85] Mack D.R., Michail S., Wei S., McDougall L., Hollingsworth M.A. (1999). Probiotics inhibit enteropathogenic E. coli adherence in vitro by inducing intestinal mucin gene expression. Am. J. Physiol..

[86] Mattar A.F., Teitelbaum D.H., Drongowski R.A., Yongyi F., Harmon C.M., Coran A.G. (2002). Probiotics up-regulate MUC-2 mucin gene expression in a Caco-2 cell-culture model. Pediatr. Surg. Int..

[87] Caballero-Franco C., Keller K., De Simone C., Chadee K. (2007). The VSL#3 probiotic formula induces mucin gene expression and secretion in colonic epithelial cells. Am. J. Physiol. Gastrointest. Liver Physiol..

[88] Gaudier E., Michel C., Segain J.P., Cherbut C., Hoebler C. (2005). The VSL#3 probiotic mixture modifies microflora but does not heal chronic dextran-sodium sulfate-induced colitis or reinforce the mucus barrier in mice. J. Nutr..

[89] Mashimo H., Wu D.C., Podolsky D.K., Fishman M.C. (1996). Impaired defense of intestinal mucosa in mice lacking intestinal trefoil factor. Science.

[90] Podolsky D.K., Lynch-Devaney K., Stow J.L., Oates P., Murgue B., DeBeaumont M., Sands B.E., Mahida Y.R. (1993). Identification of human intestinal trefoil factor. Goblet cell-specific expression of a peptide targeted for apical secretion. J. Biol. Chem..

[91] Suemori S., Lynch-Devaney K., Podolsky D.K. (1991). Identification and characterization of rat intestinal trefoil factor: Tissue- and cell-specific member of the trefoil protein family. Proc. Natl. Acad. Sci. USA.

[92] Hoffmann W. (2005). Trefoil factors TFF (trefoil factor family) peptide-triggered signals promoting mucosal restitution. Cell. Mol. Life Sci..

[93] Kindon H., Pothoulakis C., Thim L., Lynch-Devaney K., Podolsky D.K. (1995). Trefoil peptide protection of intestinal epithelial barrier function: Cooperative interaction with mucin glycoprotein. Gastroenterology.

[94] Li J., Zhou R., He W.C., Xia B. (2011). Effects of recombinant human intestinal trefoil factor on trinitrobenzene sulphonic acid induced colitis in rats. Mol. Biol. Rep..

[95] Vandenbroucke K., Hans W., Van Huysse J., Neirynck S., Demetter P., Remaut E., Rottiers P., Steidler L. (2004). Active delivery of trefoil factors by genetically modified Lactococcus lactis prevents and heals acute colitis in mice. Gastroenterology.

[96] Kim M.H., Kim H. (2017). The Roles of Glutamine in the Intestine and Its Implication in Intestinal Diseases. Int. J. Mol. Sci..

[97] Wang Z.E., Wu D., Zheng L.W., Shi Y., Wang C., Chen Z.H., Peng X. (2018). Effects of glutamine on intestinal mucus barrier after burn injury. Am. J. Transl. Res..

[98] Wu D., Su S., Zha X., Wei Y., Yang G., Huang Q., Yang Y., Xia L., Fan S., Peng X. (2023). Glutamine promotes O-GlcNAcylation of G6PD and inhibits AGR2 S-glutathionylation to maintain the intestinal mucus barrier in burned septic mice. Redox Biol..

[99] Furness J.B. (2012). The enteric nervous system and neurogastroenterology. Nat. Rev. Gastroenterol. Hepatol..

[100] Yang D., Jacobson A., Meerschaert K.A., Sifakis J.J., Wu M., Chen X., Yang T., Zhou Y., Anekal P.V., Rucker R.A. (2022). Nociceptor neurons direct goblet cells via a CGRP-RAMP1 axis to drive mucus production and gut barrier protection. Cell.

[101] Pelaseyed T., Bergstrom J.H., Gustafsson J.K., Ermund A., Birchenough G.M., Schutte A., van der Post S., Svensson F., Rodriguez-Pineiro A.M., Nystrom E.E. (2014). The mucus and mucins of the goblet cells and enterocytes provide the first defense line of the gastrointestinal tract and interact with the immune system. Immunol. Rev..

[102] Gustafsson J.K., Johansson M.E.V. (2022). The role of goblet cells and mucus in intestinal homeostasis. Nat. Rev. Gastroenterol. Hepatol..

[103] Johansson M.E., Larsson J.M., Hansson G.C. (2011). The two mucus layers of colon are organized by the MUC2 mucin, whereas the outer layer is a legislator of host-microbial interactions. Proc. Natl. Acad. Sci. USA.

[104] Narula N., Wong E.C.L., Dehghan M., Mente A., Rangarajan S., Lanas F., Lopez-Jaramillo P., Rohatgi P., Lakshmi P.V.M., Varma R.P. (2021). Association of ultra-processed food intake with risk of inflammatory bowel disease: Prospective cohort study. BMJ.

[105] Armstrong H.K., Bording-Jorgensen M., Santer D.M., Zhang Z., Valcheva R., Rieger A.M., Sung-Ho Kim J., Dijk S.I., Mahmood R., Ogungbola O. (2023). Unfermented beta-fructan Fibers Fuel Inflammation in Select Inflammatory Bowel Disease Patients. Gastroenterology.

[106] Bosscher D., Van Caillie-Bertrand M., Van Cauwenbergh R., Deelstra H. (2003). Availabilities of calcium, iron, and zinc from dairy infant formulas is affected by soluble dietary fibers and modified starch fractions. Nutrition.

[107] Singh V., Yeoh B.S., Walker R.E., Xiao X., Saha P., Golonka R.M., Cai J., Bretin A.C.A., Cheng X., Liu Q. (2019). Microbiota fermentation-NLRP3 axis shapes the impact of dietary fibres on intestinal inflammation. Gut.

[108] Zheng Y., Zhang Z., Tang P., Wu Y., Zhang A., Li D., Wang C.Z., Wan J.Y., Yao H., Yuan C.S. (2023). Probiotics fortify intestinal barrier function: A systematic review and meta-analysis of randomized trials. Front. Immunol..

